# Racial Discrimination from Patients: Institutional Strategies to Establish Respectful Emergency Department Environments

**DOI:** 10.5811/westjem.2021.3.51582

**Published:** 2021-07-14

**Authors:** Anita Nandkumar Chary, Mariam Olivia Fofana, Harajeshwar Singh Kohli

**Affiliations:** * Massachusetts General Hospital, Brigham and Women’s Hospital, Department of Emergency Medicine, Boston, Massachusetts; † Duke University Medical Center, Division of Emergency Medicine, Durham, North Carolina

## INTRODUCTION

Social identity-based discrimination from patients against healthcare providers is a prevalent and well-documented phenomenon.^1^–^3^ Numerous studies and essays detail clinicians’ experiences of slurs, harassment, and violence from patients based on racial identity.^4^–^8^ In this essay, we advance arguments about how emergency departments (ED) should respond to interpersonal racism from patients. We use an anthropological definition of race as a socially constructed way of categorizing humans based on perceived physical traits, such as skin and hair color.^9^ However, race does not have an inherent biological or genetic basis: there is greater physical and genetic variation within racial groups than between them, and racial categories vary across societies.^9^ Rather, race is assigned in ways that afford privilege, wealth, and power to some, while disadvantaging others.^9^,^10^

In this editorial, we focus on interpersonal racism, defined as the expression of racial discrimination between individuals, including racial jokes, harassment, and singling someone out on the basis of race.^10^ We recognize that racial discrimination can manifest in more subtle ways, such as microaggressions, or commonplace verbal or behavioral exchanges that convey hostility—often unintentionally—toward marginalized groups.^11^ Given significant variability in healthcare providers’ recognition and acceptance of microaggressions as discriminatory,^12^ our advocacy here focuses on unified institutional responses to interpersonal racism. We are interested in increased discussion about protecting the rights and wellbeing of emergency physicians at the same time that we address patients’ medical needs, particularly in our climate of profound political polarization in the United States.

### Strategies for Dealing with Racist Patients: the Lens from Acute Care Settings

Biomedical scholarship predominantly advances the individual physician’s appeasement, negotiation, and accommodation of racist patients, with a focus on prioritizing and moving forward a patient’s medical care.^13^,^14^ For example, when a patient declines care from a physician who is a racial minority, hospital staff often seek out another physician to care for the patient.^4^ When a patient yells racial slurs at physicians or tells them to “go back to their country,” the physician is expected to respond to the patient courteously, if at all, in the interest of maintaining professionalism,^6^ or to re-orient themselves to patient needs and “depersonalize” their experiences.^15^ These strategies construe acceptance of racism from patients as necessary to maintain the therapeutic relationship and imply that the targets of such abuse should be willing to incur it as part of the inevitable costs of the job. However, as seen in the response to sexual discrimination and harassment and bullying, both in broader society and in the medical profession specifically, attitudes and behaviors that were once accepted as part of the prevailing culture are increasingly and rightfully being denounced.^16^,^17^ Recognition of the detrimental effects of sexual discrimination and bullying, including psychological consequences, hindered career advancement, and the effects of burnout and attrition on the profession as a whole, have led organizations such as the Joint Commission on Accreditation of Healthcare Organizations and the National Academies of Sciences, Engineering and Medicine to call for institutional and systemic responses.^18^,^19^

Less emphasis has been placed on institutional responses to interpersonal racism in healthcare settings. Williams and Rohrbaugh suggest conceptualizing racist language as *verbal assault* to underscore traumatic consequences and to trigger reporting of such encounters to administrators, as is done for physical assaults that occur in hospitals.^4^ They also suggest team debriefing and de-escalation trainings to help cope with disruptive and discriminatory patients.^4^ Others have advocated for involvement of ethics committees with disruptive and hateful patients.^13^

Unique aspects of emergency care settings affect the possibilities for individual and institutional responses to interpersonal racism. Prior evidence suggests that workplace violence is more common in EDs than in other clinical settings, yet emergency physicians may feel ill-equipped and unjustified in responding to racist abuse from patients who are experiencing an acute psychiatric crisis, delirium, intoxication, or are otherwise in distress.^20^ Unlike longitudinal care settings, the ED leaves little time for clinicians to establish a therapeutic relationship with patients, which may further disincentivize confronting racist patients. Emergency physicians also face pressure to appease racist patients due to the Emergency Medical Treatment and Labor Act (EMTALA), which stipulates that all patients who seek care in the ED must receive a medical screening examination and stabilization of an emergency medical condition, regardless of their social identity, ability to pay, or behavior.^21^ Additionally, time constraints, acuity, and frequent changes in team composition preclude emergency clinicians’ abilities to acutely or consistently involve ethics committees, debrief in real time, or find another clinician to care for a racist patient.

Consider the following scenarios:

#### Scenario

A Black emergency medicine resident begins a primary survey during a trauma resuscitation. The patient, who is alert, shouts racial slurs at the resident, including “[N-word] bitch,” and demands another physician. None of the team members present acknowledge the discriminatory behavior and proceed with the rest of the survey.

#### Scenario

A Sikh attending emergency physician evaluates a young intoxicated male patient cursing at staff from the stretcher. When the patient sees the physician, who wears a turban, he begins yelling, “I don’t want to see a foreign doctor! I want to see an American doctor!”

In each case, the physician is emotionally traumatized by the hateful remarks, but may feel morally and legally compelled to evaluate the patient for an emergency medical condition warranting stabilization. If the physician determines that the patient does have an emergency medical condition requiring treatment, then we see three viable, but imperfect, options. First, the physician can continue treating the patient, assuming the patient allows, prioritizing the patient’s health needs over the physician’s own emotional wellbeing, and despite the likelihood of a poor therapeutic alliance. Second, if not in a single coverage ED, the physician could ask another physician, if available, to care for the patient. Third, the physician can supervise and direct the patient’s care through an intermediary–a resident physician, advanced practice provider, nurse, or technician—acknowledging that this could lead to variations in care.

The identity of each physician, encompassing their personal values, experiences, and social and emotional capital, also affects their potential immediate responses. In the first scenario, the trainee, who lacks support from the team, does not have the power to excuse herself from the care of the patient. Furthermore, the trainee may fear repercussions of reporting the incident, such as being seen as too emotionally sensitive, unable to prioritize patients’ needs, or stereotyped as an angry minority. In the second scenario, the attending physician may feel compelled to compartmentalize the interaction in the moment and maintain composure as the leader of the care team, particularly if concerned about an emergency medical condition.

These scenarios highlight that no singular prescriptive practice can be recommended for emergency physicians who experience interpersonal racism from patients. These physicians should not be charged with personally responding to these situations if they do not desire to do so. Rather, they would benefit from broader institutional support and anti-racist policies as below.

### Suggested Institutional Actions to Establish Respectful Work Environments

We suggest three critical institutional actions that EDs should take to respond to interpersonal racism from patients and establish respectful work environments. First, EDs should establish a patient, visitor, and staff code of conduct. An ED code of conduct should clearly state that discriminatory language and behaviors are not tolerated (see Figure). The code of conduct should be displayed in view of patients and visitors and be physically and electronically accessible to staff as other policies are. If an individual displays discriminatory language or behaviors, staff should provide a verbal reminder of the code of conduct. If the individual then persists in racist language or behaviors, the care team should assess the individual’s ability to be discharged. EMTALA and its mandate originated from the guiding principle to care for indigent and uninsured patients. If a racist and disruptive patient does not have a medical condition requiring emergency stabilization and could otherwise be treated as an outpatient, discharging the patient is acceptable. An individual’s right to and need for healthcare must be weighed against a clinician’s safety and right to work in an environment free from discrimination. While the First Amendment protects hate speech up until it incites violence,^22^,^23^ employers are proscribed by Title VII of the Civil Rights Act (CRA) of 1964 from engaging in employment discrimination practices.^24^ A code of conduct created and promulgated by a hospital is a measure that can promote an environment that is firmly anti-racist and anti-discrimination.

Second, EDs should establish expectations that staff, as members and representatives of the institution, can and should address discrimination from patients in real time. Immediate responses to racism can be particularly meaningful and supportive if expressed by a bystander, rather than the target.^25^ A bystander response should ideally both address the inappropriateness of racist behavior and lend support to the target of racism.^26^ Hospital staff who witness discrimination should explicitly make a statement such as this: “Discrimination is not acceptable in the hospital environment”; or “Racist remarks are not tolerated in our emergency department” (see Table). Regardless of a patient’s or visitor’s mental status, staff should remind them of the code of conduct, as some individuals with mild intoxication and psychiatric illness are redirectable.

Lending support to the target of discrimination may take the form of an individual check-in with the target, such as, “I’m sorry that happened. How can I support you?” A short staff debrief establishing that interpersonal racism is not acceptable can unify the team and express alliance with the target. While immediate debriefing may not be feasible in all high-volume and high-acuity situations, making the time to do so, even if quickly, contributes to a workplace environment of solidarity. Additionally, the transition of care of a discriminatory patient, who still requires treatment, to another physician is in itself a powerful act. This is fundamentally different than acquiescing to racist patients’ demands: the decision ultimately rests with the victim, and the intent is to protect them from further abuse. This can be achieved through a protocol that is disseminated and discussed among the physicians in a group and that can be referenced and activated in real time.

While we acknowledge limitations of such protocols in single-coverage EDs as well as situations where patients lack capacity or have immediately life-threatening illness, leadership should foster a culture that normalizes and promotes this form of support whenever feasible. Establishment of these expectations and guidance on how staff can respond to racism can be offered in the form of an announcement at a staff meeting, an email, or, where resources are available, through formalized bystander training.^27^ Sample language is outlined in the Table.

Third, EDs should create or link to hospital-wide incident reporting mechanisms. There is a clear precedent for healthcare organizations to implement systemic interventions to prevent and report physical assaults in the workplace.^28^ Incident reporting, whether to department leadership, human resources, anti-racism committees, and/or institutional centers for diversity and inclusion, could contribute to administrative knowledge about the frequency and scope of racist encounters. Additionally, as immediate staff debriefing may not occur in emergency care settings, reporting mechanisms could facilitate a third party reaching out to and supporting the targeted clinician after a racist encounter.

Patients who commit physical aggression against hospital staff receive flags in their charts, leading to warning notifications upon opening the electronic health record. We suggest implementing similar electronic warning systems for patients who engage in racist verbal aggression. Repeat offenders may have a contract or care plan developed, clearly outlining behavioral expectations when receiving emergency and hospital-based care.

In this essay, we focus on race, recognizing the difficulty and awkwardness of conversations about racism when compared to other forms of social identity-based discrimination. However, our recommendations can just as easily apply to creating institutional support for those marginalized on the basis of other identities, such as gender, sexual orientation, or ability status.

## CONCLUSION

Institutional responses to interpersonal racism can empower emergency physicians to address discrimination from patients in real time. Rather than relying solely on targets of racial discrimination to accommodate or directly respond to patients, we advocate for institutional responses to promote respectful and supportive workplace environments.

## Figures and Tables

**Figure:**
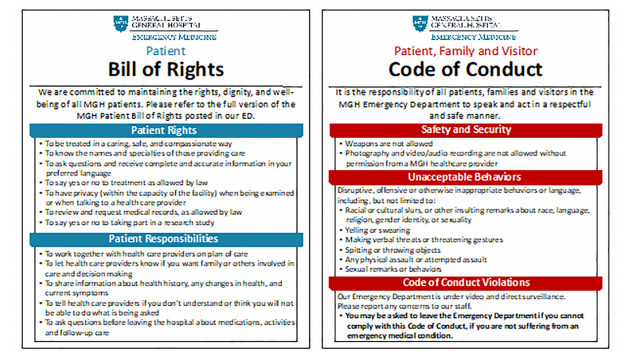
Code of Conduct, Massachusetts General Hospital Emergency Department. Used with permission from the Department of Emergency Medicine, Massachusetts General Hospital.

**Table:** Sample language for addressing interpersonal racism from patients. Developed in collaboration with the Social Emergency Medicine Interest Academy of the Harvard Affiliated Emergency Medicine Residency

Situation	Sample language	Strategies employed
Bystander outlines behavioral expectations for patient or visitor	“Racist language is not acceptable in our hospital. Please be respectful.” “I must remind you that our code of conduct outlines that discriminatory language and behavior is not tolerated.” “Racist remarks are not tolerated in our emergency department. Please remember that as we take great care of you.” “We are doing our best to take excellent care of you. Please refrain from making racist statements.”	Rely on institutional policy to strengthen position Take firm but professional approach Remind patient/family of therapeutic intent
Bystander checks in with target	“I am sorry that happened. It upset me. I wanted to check in on how you are doing.” “I am sorry that happened. Please let me know how I can support you.” “I am sorry that happened. I would like to report this incident to our supervisors, if that is okay with you.”	Acknowledge situation, name own feelings without projecting them onto target, offer support
Care team member leads debrief	“Our patient’s racist language and behaviors today are not acceptable. I’d like to remind everyone of our code of conduct.”	Outline interpersonal racism as not tolerated Remind staff of institutional policy
Care team member assists with provider transition of care when a physician has experienced interpersonal racism	[to colleague:] “I am sorry about what happened. I am willing to assume care of this patient.” “This patient has been stabilized and it is appropriate for their care to be handed off.” “We can have another provider take care of this patient primarily.” [to trainee:] “I’d like to have another provider take care of this patient primarily. You did nothing wrong, but I don’t think it is a positive environment for you to remain in.”	Acknowledge situation and offer alternative Affirm appropriateness of care handoff Recognize that victims of interpersonal racism, particularly trainees, may not feel empowered to voice a preference to not participate in the care of discriminatory patients
